# The SF12 Well in LoRaWAN: Problem and End-Device-Based Solutions

**DOI:** 10.3390/s21196478

**Published:** 2021-09-28

**Authors:** Lluís Casals, Carles Gomez, Rafael Vidal

**Affiliations:** Department of Network Engineering, Universitat Politècnica de Catalunya, 08860 Castelldefels, Spain; carlesgo@entel.upc.edu (C.G.); rafael.vidal@entel.upc.edu (R.V.)

**Keywords:** LoRaWAN, LPWAN, SF12 well, congestion, well fall time, AFLoRa

## Abstract

LoRaWAN has become a popular technology for the Internet of Things (IoT) device connectivity. One of the expected properties of LoRaWAN is high network scalability. However, LoRaWAN network performance may be compromised when even a relatively small number of devices use link-layer reliability. After failed frame delivery, such devices typically tend to reduce their physical layer bit rate by increasing their spreading factor (SF). This reaction increases channel utilization, which may further degrade network performance, even into congestion collapse. When this problem arises, all the devices performing reliable frame transmission end up using SF12 (i.e., the highest SF in LoRaWAN). In this paper, we identify and characterize the described network condition, which we call the SF12 Well, in a range of scenarios and by means of extensive simulations. The results show that by using alternative SF-management techniques it is possible to avoid the problem, while achieving a packet delivery ratio increase of up to a factor of 4.7.

## 1. Introduction

Low Power Wide Area Network (LPWAN) technologies have gained significant momentum as wireless connectivity solutions for enabling the Internet of Things (IoT) applications. LPWAN technologies are typically based on the star topology paradigm, whereby the IoT devices communicate directly with neighboring radio gateways. This feature, along with the support of long range (in the order of kilometers), offers reduced infrastructure cost and complexity compared with short-range multihop topologies. In addition, LPWAN technologies provide low energy consumption, enabling multiyear lifetimes for battery-operated devices. However, the advantages of LPWAN technologies come at the expense of challenging constraints in terms of message sizes, bit rates, and message rates. These unique features of LPWAN technologies have attracted the attention of industry, academia, and standards development organizations [1,2,3,4].

One of the most prominent LPWAN technologies is LoRaWAN [5]. Two of the main reasons for the popularity of this technology are the following: (i) It is based on the sub-GHz unlicensed spectrum, which facilitates adoption, even if it also involves duty cycle constraints due to spectrum access regulations in some world regions; and (ii) it is possible to deploy a private LoRaWAN network without the need to rely on third-party entities (e.g., network operators).

Since the initial specification of LoRaWAN was published, many researchers have devoted their efforts to investigating the performance of this technology [1,5,6,7,8,9,10,11]. However, LoRaWAN deployments are often still in the relatively initial stages, and network performance may become compromised as the number of connected end-devices increases, even for a relatively low number of such devices.

In this paper, we identify and illustrate a problem which we call the *Spreading Factor 12 (SF12) Well* and evaluate a number of solutions to counter it. LoRaWAN offers optional link-layer reliability, based on link-layer acknowledgments and retransmissions. When acknowledgments are not received by a sender after transmitting a frame in reliable mode, the sender typically tends to reduce its physical layer bit rate. However, this reaction increases uplink and, even worse, downlink channel utilization. As a result, network performance may degrade steadily into congestion collapse. In this paper, we characterize the SF12 Well problem in a range of scenarios, and we show that it is possible to mitigate it, maintaining good performance as the offered load increases by using different SF management techniques.

In order to carry out the study, we developed and used a simulator called Advanced Framework for LoRa (AFLoRa), a LoRaWAN simulation environment that uses FLoRa [12] as a basis, albeit with significant enhancements and additions. As a side-contribution of this paper, we offer the simulator publicly [13].

The remainder of the paper is organized as follows. Section 2 presents the main features of LoRaWAN, focusing on the parameters and mechanisms most relevant to the present work. Section 3 reviews related work. Section 4 illustrates the SF12 Well problem in different scenarios in terms of the offered load and the ratio of acknowledged traffic. Section 5 evaluates a number of techniques intended to counter the SF12 Well problem, showing their performances and trade-offs. Finally, Section 6 provides the main remarks from this work.

## 2. LoRaWAN Main Features

This section presents the main characteristics of LoRaWAN, focusing on the features and functionality which are the most relevant in the context of this paper. The section comprises three subsections. The first one gives a LoRaWAN overview, whereas the remaining two subsections focus on the functionality supported by LoRaWAN at its two lowest layers, i.e., the physical layer and the MAC layer, respectively.

### 2.1. LoRaWAN Overview

LoRaWAN is a wireless technology that offers long range (often in the order of kilometers) [6] while supporting low energy consumption (e.g., allowing multiyear lifetime for battery-operated devices) [14]. As in other LPWAN technologies, long range is achieved at the expense of reduced communication capacity. However, this feature does not pose a problem for many IoT use cases.

**Figure 1:**
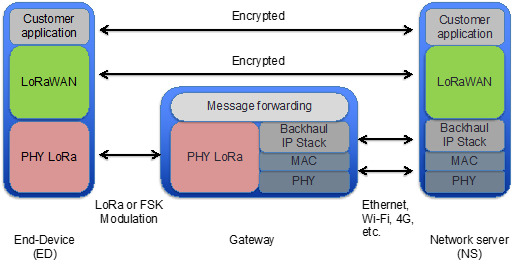
LoRaWAN protocol stack for each network element.

The LoRaWAN network architecture comprises three main types of network entities: end-devices (EDs), gateways, and a network server (NS). These elements are organized in a topology known as the *star of stars* [15]. The EDs typically correspond to constrained devices such as sensors. The EDs transmit LoRaWAN messages to the NS as the destination through one or more gateways. This type of message transmission is known as uplink transmission. In the opposite direction, i.e., in the downlink, the NS may transmit LoRaWAN messages to the EDs through only one gateway. Communication between the EDs and the gateways is carried out by means of a physical layer called LoRa. The gateways and the NS are connected by means of an IP-based network, while the gateways forward LoRaWAN messages between the EDs and the NS (Figure 1) [14].

LoRaWAN defines three classes in terms of supported features and functionality: class A, class B, and class C. Class A, which is also referred to as basic LoRaWAN, is mandatory for all LoRaWAN devices. In this class, and for the sake of energy saving, downlink transmission is only allowed in time intervals called receive windows, which are subsequent to an uplink transmission. Class B is defined on the basis of class A, offering additional downlink transmission opportunities at times which may be scheduled a priori. In contrast with class A and class B, class C offers no constraints for downlink transmission. However, the class C EDs cannot turn off their radio interface and are not suitable for devices with a constrained energy source. Most LoRaWAN devices implement only class A features as the other classes are optional. 

This paper assumes that class A is used in a LoRaWAN network. The next two subsections overview the main features of the class A LoRaWAN physical layer and the MAC layer, respectively.

### 2.2. LoRaWAN Physical Layer

LoRaWAN supports two types of modulations for physical transmission between an ED and a gateway: the LoRa modulation and the Gaussian Frequency Shift Keying (GFSK). The former is the most frequently used modulation in many scenarios. LoRa is based on a chirp spread spectrum [16]. The duration of a LoRa symbol depends on the Spreading Factor (SF) in use, as a LoRa symbol comprises 2^SF^ chirps [6]. Six SFs (ranging from seven to twelve) are defined, leading to six different corresponding Data Rates (DRs). The SFs are orthogonal, which contributes to spectral efficiency.

LoRaWAN has been defined to support operation in several world regions. In our study, we consider the LoRaWAN physical layer characteristics specified for the European Union (EU), including the use of the 868 MHz band, where three default radio channels are defined: 868.10 MHz, 868.30 MHz, and 868.50 MHz. These channels are characterized by a bandwidth of 125 kHz, use the LoRa modulation, and offer several data rates, from DR0 to DR5, which correspond to 0.3 kbps to 5 kbps, respectively (see Table 1). DR6 and DR7 are optional (the latter is the only one based on the GFSK). In order to provide robustness to the communication, frequency channel hopping is used.

ETSI regulations establish spectrum access restrictions on the duty cycle, including a duty cycle limitation to less than 1% for the band between 868.0 MHz and 868.6 MHz. LoRaWAN complies with the mentioned duty cycle restriction by introducing an idle interval of suitable duration after the transmission of a message. 

**Figure 2:**
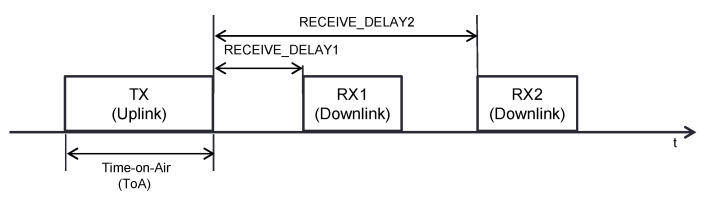
Uplink transmission followed by the two receive windows, RX1 and RX2, defined in LoRaWAN class A.

In class A, message exchanges between an ED and the NS are initiated by the former. The NS is allowed to transmit only in one of two available receive time windows, called RX1 and RX2 (see Figure 2), which are available after a message transmission by the ED. RX1 and RX2 start after RECEIVE_DELAY1 and RECEIVE_DELAY2 from the end of the uplink transmission, with default values for these parameters of 1 s and 2 s, respectively. If the NS has to transmit a downlink message, such a transmission will only be possible when a new receive window is open. By default, the DR used in RX1 is the same as the DR of the last uplink message transmission. In RX2, DR0 (i.e., SF12) is used by default, on the 869.525 MHz frequency band [17]. This band is dedicated to the downlink, and it is subject to a duty cycle limitation of less than 10% [18].

### 2.3. LoRaWAN MAC Layer

LoRaWAN supports optional reliability, by means of positive acknowledgments (ACKs) and MAC layer retries. For each data frame, an ED may indicate whether an ACK is requested or not. For a message requiring an ACK (also called a *confirmed* frame), the ACK may be received in one of the next two receive windows. If the ACK is not received, the ED retransmits the same frame up to a maximum number of retries. In most currently deployed EDs [19,20], the first two transmission attempts use the same DR and, thereafter, the DR decreases to the next lower DR every two attempts (unless DR0 is used). Once the maximum number of transmission attempts for the same frame is reached, confirmed transmission failure is notified to the application layer. Each retransmission is performed after a timeout, which is initiated at the start time of the last second receive window, with a duration chosen randomly between 1 and 3 s, by default [17].

The LoRaWAN MAC layer also defines the Adaptive Data Rate (ADR) mechanism, which allows the NS to control the ED parameters, such as the DR, the transmit power, and the channels used, based on the estimated link quality.

## 3. Related Work

Previous work has pointed out that confirmed traffic in LoRaWAN may be impaired by DC restrictions that limit downlink capacity, rendering gateways as bottlenecks that cause packet delivery ratio decrease [7,8,9,21,22,23]. However, the consequent problem of congestion collapse remains unexplored as of this writing. 

The gateway bottleneck issue aggravates as the SF used by the devices increases, where using SF12 corresponds to the worst scenario. SF12 actually offers the best link range in LoRaWAN [6,24]. However, such an advantage is achieved at the expense of minimizing frame payload size and bit rate, which increases frame Time-on-Air (ToA) [14], the ED power consumption [14], and the uplink collision probability [10]. In the downlink, as aforementioned, a gateway uses the same SF as the one used in the uplink in RX1, and SF12 in RX2 [17]. Hence, when the use of SF12 by the EDs increases, the use of SF12 by the gateways for downlink transmission (e.g., to send ACKs) increases as well. In turn, this behaviour minimizes the downlink capacity of the gateways [11], which may result in congestion losses [9].

Different proposals intended to mitigate the bottleneck problem involve the use of multiple gateways [23], ACK aggregation (i.e., grouping ACK information in fewer, longer-sized ACKs) [25], and sending a single ACK to a group of EDs [26]. The first type of solution obviously increases network cost, whereas the other mentioned solutions require major MAC layer modifications, affecting both communication endpoints: the EDs and the NS.

In order to minimize the negative impact of SF12, one possible approach could be the leveraging of the ADR mechanism [15,19,27]. Only recommended when propagation conditions are stable, the ADR aims to optimize the uplink data rate, air time, and energy consumption by using the ED link budget as the input. Many research works propose ADR optimizations [28,29,30,31,32,33]. Such optimized ADR schemes focus on different main objectives, such as throughput improvement [29,30], energy efficiency [31,32], or scalability [33].

In [29], a new ADR algorithm called EXPLoRa-C is proposed and evaluated in terms of throughput by means of theorical analysis and simulations and taking into account the capture effect. On the other hand, in [30] a scheme for power transmission and SF allocation is presented to achieve a fairer network operation by optimizing the packet error rate for nodes far from the gateway. Energy efficiency is an objective in [32], where an analytical model is developed to select the SF, channel width, and code rate to optimally satisfy a given Quality of Service (QoS). In [31], the authors implement an improved ADR scheme that yields a better data extraction ratio and a lower number of collisions, while keeping energy consumption. On the other hand, the authors in [33] propose an improved ADR mechanism that increases reliability and energy efficiency fairly for any network size and increases the delivery ratio in dense networks.

However, the ADR ignores possible congestion problems. In addition, the ADR relies on the exchange of MAC commands between the EDs and the NS. Thus, its usage adds more pressure to the downlink bottleneck and is inefficient in scenarios with a high number of packet losses [8]. Another important drawback of the ADR is its data rate backoff sequence [21]. In fact, the literature highlights that this mechanism was designed for coverage problems, and it fails in the presence of congestion [17]. However, the authors of these works do not investigate this area further.

A modified ADR scheme, intended to be congestion-aware, has been proposed in [34]. However, this proposal exhibits the following drawbacks: (i) It requires all network traffic to be confirmed as ACKs are used for congestion estimation; and (ii) it is based on too simple an analytical model, where the DC restrictions are not considered, and the number of losses due to congestion is assumed to have a constant value, for which, in addition, no motivation is given.

The works reviewed above are based on simulation (with one exception [22]). There also exists a wide body of LoRaWAN studies focused on experimental or real-application scenarios [35,36,37,38,39]. Some of these works investigate LoRaWAN features that are relevant regarding network performance. The authors in [35] study the use of different DR settings at the edge of link quality and as a result propose using higher DR settings, even when packet delivery probability is lower, for the sake of a greater effective bit rate. Another work reports the performance of a public LoRaWAN deployment; the authors of this work collect the Signal-to-Noise Ratio (SNR) and Received Signal Strength Indication (RSSI) values to determine maximum network coverage and the SF distribution of the EDs [36]. Other works focus rather on the feasibility of specific applications or deployments. For example, the authors in [37] explore the viability of a sailing monitor system based on LoRa, while another study measures the performance a LoRa link can achieve in underground to aboveground communications for different types of soil [38]. Another study evaluates methods for LoRaWAN coverage survey in the context of a smart campus [39]. However, no empirical work investigates the congestion collapse issues in LoRaWAN.

Based on the literature review, we conclude that, to the best of our knowledge, the present paper is the first one that shows and characterizes the SF12 Well problem. In addition, the paper presents and compares several ED-based solutions, showing how this problem can be mitigated without involving the NS.

## 4. The Problem

As aforementioned, confirmed traffic in LoRaWAN may lead to retransmissions that progressively increase the SF values used by the EDs performing confirmed transmissions. Eventually, all such EDs will end up using SF12. This network condition, which we call the SF12 Well, results in an inefficient network operation due to the high frame ToA. The SF12 Well can lead to severely degraded network performance, even for a relatively low offered load.

In this section, we characterize the SF12 Well problem by means of extensive simulations. We evaluate a diversity of scenarios in order to determine the conditions that cause and accelerate the SF12 Well. In addition, we illustrate the performance degradation produced by the SF12 Well. For the evaluation, we developed a simulator called AFLoRa on the basis of the FLoRa simulator.

This section is divided into four parts. Section 4.1 presents the main features of the AFLoRa simulator. Section 4.2 provides the details of the scenarios considered in the evaluation, along with the simulation conditions assumed. Section 4.3 demonstrates the SF12 Well problem. Finally, we show and discuss a set of global performance results in Section 4.4.

### 4.1. Simulator Details

In this section, we highlight the most important features of the simulator that we developed and used. Our simulator, called AFLoRa [13], is based on a LoRaWAN simulation environment called Framework for LoRa (FLoRa) [12], albeit with significant updates and enhancements.

FLoRa was built for the OMNeT++ network simulator by reusing components from the INET framework of the latter. FLoRa includes the main components in a LoRaWAN network, covering from physical-layer to application-layer aspects. It follows the main details of the LoRaWAN specification for class A EDs with unconfirmed transmission mode. The model used in the FLoRa implementation includes collisions and the capture effect at the physical layer and end-to-end communication between the EDs and the NS through a backhaul network [33].

However, FLoRa does not include some important LoRaWAN network characteristics, such as confirmed transmission mode, transmission queue, losses due to full queue, and duty cycle restrictions for both the EDs and the gateway, among others. For this reason, we updated the original FLoRa simulator for the present work, leading to an enhanced simulator that we call AFLoRa. Our simulator provides a more complete implementation of the LoRaWAN specification, with confirmed transmissions, restrictions due to duty cycle regulations, and a queuing mechanism for the application layer and the MAC layer. AFLoRa also supports the alternative SF management modes considered in this paper as possible techniques to counter the SF12 Well problem (see Section 5), along with the default LoRaWAN behavior.

Furthermore, we also fixed and/or improved several implementation details of the simulator. We corrected the RX2 time scheduling, and we fixed the idle-time values required to comply with the duty cycle restrictions. Moreover, we reorganized the original implementation of the application and the MAC layers to better match the structure described in the LoRaWAN specification, with a stronger separation of those layers, which allows an easier implementation of the new features. This action also includes an update of the message structure used at the MAC and applications layers. 

### 4.2. Simulated Scenarios 

**Figure 3:**
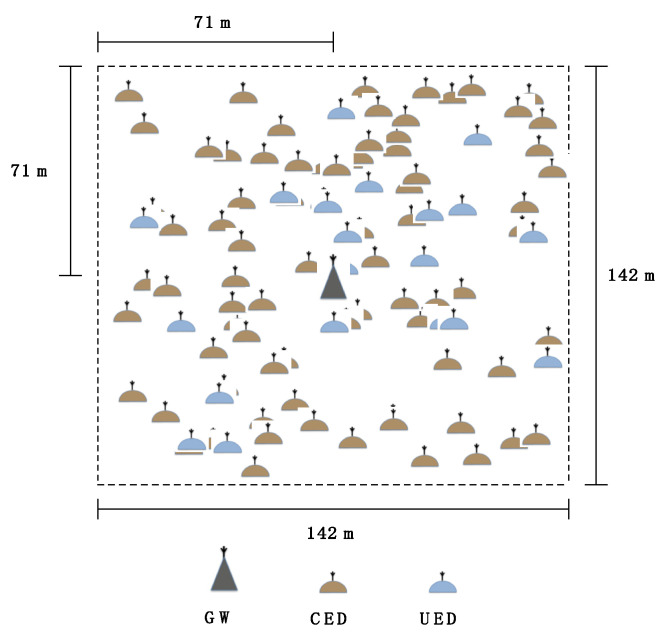
Example of EDs and gateway spatial distribution on the scenario area, where there are 80 CEDs.

In the simulations, a LoRaWAN network comprises 100 EDs. A subset of the EDs, which are referred to as Confirmed mode EDs (CEDs), use confirmed transmission. The rest of the EDs, called Unconfirmed mode EDs (UEDs), perform unconfirmed transmissions. The number of CEDs ranges from 30 to 100. Physical layer frames have a length of 20 and 15 bytes for data (uplink payload) and ACK (downlink payload) frames, respectively. For each ED, the time between the two consecutive packets follows an exponential distribution. Different uplink traffic loads are considered, from 0.9 packets/h to 18 packets/h. These values correspond to a wide range of mean time between consecutive packet transmissions at the application layer, from 4000 s to 200 s, respectively. All the EDs use a transmit power of 14 dBm, and their initial spreading factor is SF7, with a bandwidth of 125 kHz, which leads to an initial physical bit rate of 5470 bit/s (see Table 1). The EDs follow a uniformly random spatial distribution and are deployed on a square-shaped area of 142 × 142 m^2^, with a gateway in the middle of this area (see Figure 3). 

All the EDs are within the range of the gateway using SF7, ensuring that losses are not due to low link quality. The ADR mechanism is disabled in both the ED and the NS sides. Duty cycles of 1% and 10% are enforced for the EDs and for the gateway, respectively. We assumed a duty cycle of 10% for the gateway as a favorable, less restricted downlink scenario, which also corresponds to an alternative use of RX1 and RX2 (as proposed in [9]). As will be shown, the SF12 Well problem occurs even in such favorable conditions.

Each individual simulation is carried out for a simulated time of 86 days, which ensures that all the CEDs reach SF12 before the end of the simulation in all the considered scenarios.

### 4.3. Illustrating the SF12 Well Phenomenon

This subsection illustrates the behavior of the EDs and the network performance over time for two example cases, which correspond to the lowest and highest total offered loads considered in our study. In the first case, there are 30 CEDs, each one generating 0.9 packets/h. In the second case, there are 100 CEDs, and each ED aims to transmit 18 packets/h.

**Figure 4:**
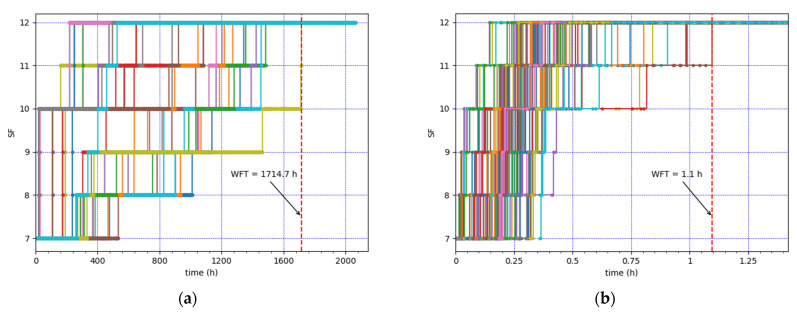
SF value used by each CED over time. (**a**) Lowest load: 30 CEDs, using an EDL of 0.9 packets/h. (**b**) Highest load: 100 CEDs, using an EDL of 18 packets/h.

Figure 4 depicts the SF value used by each node over time, for all nodes in the two described scenarios. In both cases, all the CEDs abandon the initial SF value (i.e., SF7), progressively increase the SF values used, and end up using SF12. In this paper, we define the Well Fall Time (WFT) as the time interval from the start of the network operation until the network reaches the SF12 Well state (i.e., when all the CEDs are using SF12). The WFT is equal to 1714.7 h and 1.1 h in the lowest and highest load scenarios considered (Figure 4a,b), respectively.

**Figure 5:**
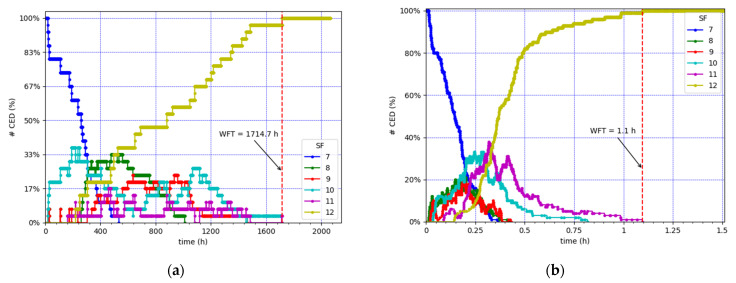
Percentage of CEDs using each SF value over time. (**a**) Lowest load: 30 CEDs, using an EDL of 0.9 packets/h. (**b**) Highest load: 100 CEDs, using an EDL of 18 packets/h.

Figure 5 shows the SF value distribution for all the CEDs over time. We can observe how, as both the number of CEDs and the EDL increase, the CEDs start using greater SF values earlier. When a high ratio (~90%) of the CEDs reach SF12, the number of EDs using SF12 increases more moderately, until all the CEDs reach SF12 at the WFT. At that time, the CEDs use the transmission medium in the most inefficient way, as SF12 leads to the highest ToA.

**Figure 6:**
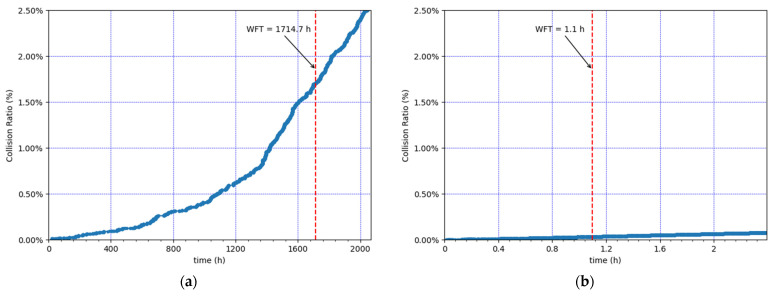
Evolution of cumulative collisions at the gateway over time. (**a**) Lowest load: 30 CEDs, using an EDL of 0.9 packets/h. (**b**) Highest load: 100 CEDs, using an EDL of 18 packets/h.

**Figure 7:**
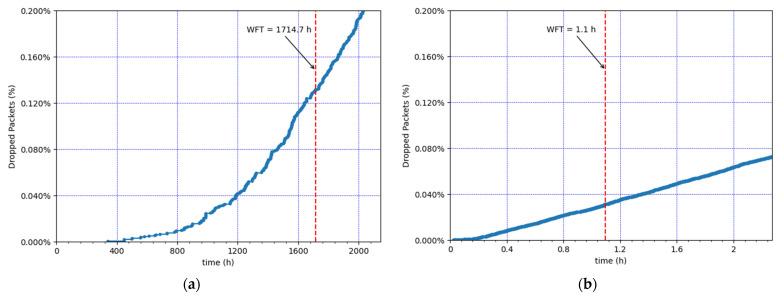
Evolution of cumulative packet drops at the EDs due to duty cycle restrictions over time. (**a**) Lowest load: 30 CEDs, using an EDL of 0.9 packets/h. (**b**) Highest load: 100 CEDs, using an EDL of 18 packets/h.

We next evaluate network performance during the considered simulated time (86 days) for both example scenarios. We first examine the evolution of the MAC frame collisions and the packet losses due to duty cycle restrictions over time (see Figure 6 and Figure 7, respectively). Figure 6 illustrates, for each given time value, the cumulative number of MAC frames that collided until that time, divided by the total number of MAC frames that were transmitted during the whole simulation time. Figure 7 depicts, for each given time value, the cumulative number of application packets that were dropped due to duty cycle restrictions, divided by the total number of application packets that were requested to be transmitted during the whole simulation time.

The results show an exponential increase in the cumulative number of frame collisions (Figure 6) and the cumulative number of application packet drops (Figure 7), approximately until the WFT. This happens because the nodes’ SF values increase, and thus the ToA increases as well. After the WFT, the cumulative number of collisions and dropped packets continues to increase, but rather linearly, since the SF values used cannot increase beyond SF12.

### 4.4. Global Results

In this subsection, we present and discuss a set of global network performance results, which are obtained for the whole set of simulation scenarios considered. The two main performance results we focus on are the WFT and the PDR. In order to better analyze network behavior, we also compute the number of collisions and the number of packet losses. Each individual result presented corresponds to the average over the 20 simulations using the same scenario configuration.

**Figure 8:**
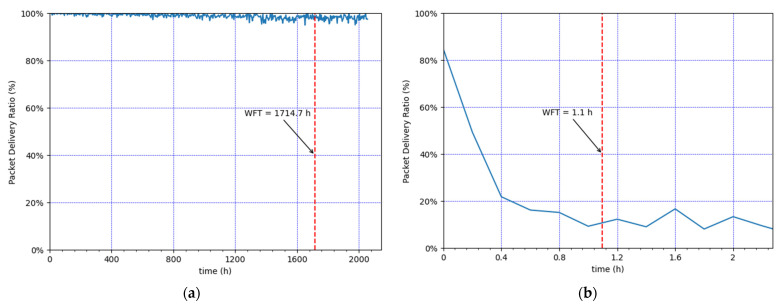
Instantaneous PDR after WFT. (**a**) Lowest load: 30 CEDs, using an EDL of 0.9 packets/h. (**b**) Highest load: 100 CEDs, using an EDL of 18 packets/h.

Finally, Figure 8 presents the instantaneous Packet Delivery Ratio (PDR) over time. Note that the instantaneous PDR corresponds to the ratio of packets received by the NS at the application layer divided by the total number of application-layer packets generated by the EDs within a relatively small interval (of 4 h and 0.2 h in Figure 8a,b, respectively) that starts at that given time. After the WFT, this performance parameter reflects the steady-state network performance. As aforementioned, in both scenarios, all the CEDs use SF12 after the WFT. However, for low loads, the PDR value after the WFT is near 100%, while in heavy load conditions the PDR is very low, around 10%. In that case, the SF12 Well problem has a dramatic impact on network performance.

**Figure 9:**
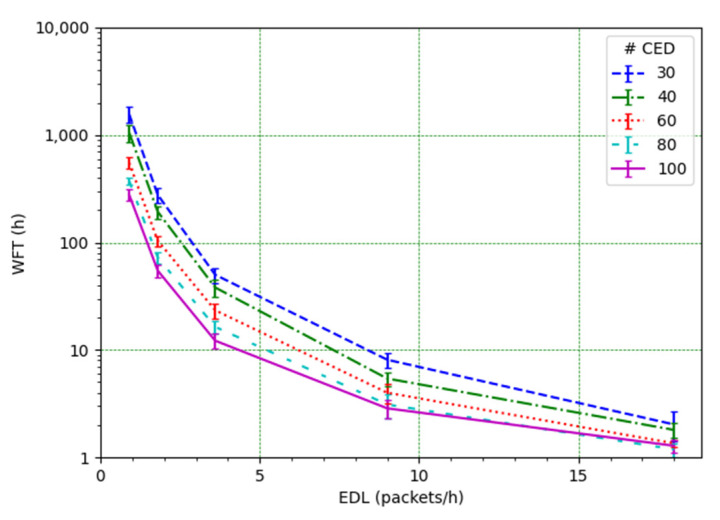
Mean and standard deviation of WFT as a function of EDL, for several numbers of CEDs.

Figure 9 shows the WFT as a function of the node load. As the offered traffic decreases, the WFT increases quickly. The greatest WFT value is obtained for 30 CEDs and EDL = 0.9 packets/h, with an average WFT equal to 65.3 days. On the other hand, the lowest WFT value is found for 100 CEDs and the EDL = 18 packets/h, with an average WFT equal to 76.7 min (and a maximum value of 93.9 min). Therefore, the WFT depends greatly on the number of CEDs and on the EDL. As the EDL decreases, the mean WFT increases very quickly (note the logarithmic scale for the vertical axis in Figure 9). The WFT differences for the different numbers of CEDs increase as well.

**Figure 10:**
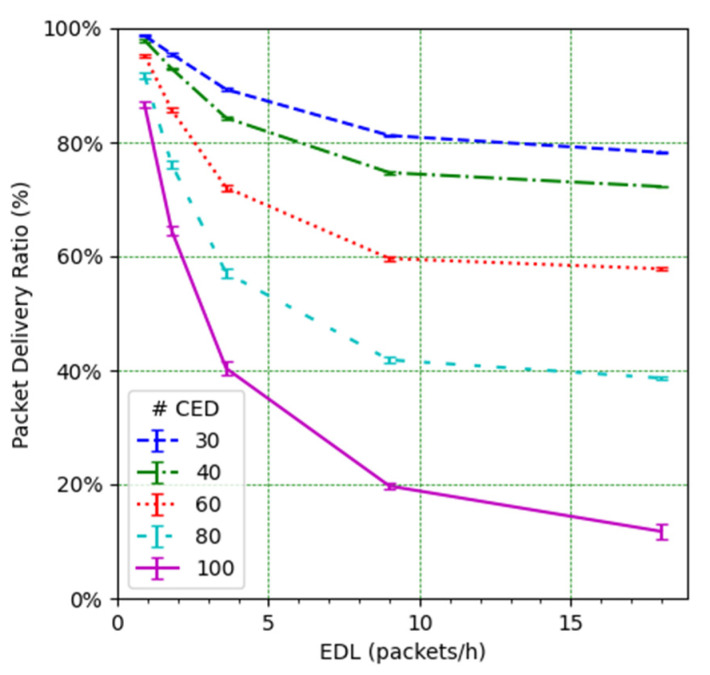
PDR vs. EDL, from all EDs.

**Figure 11:**
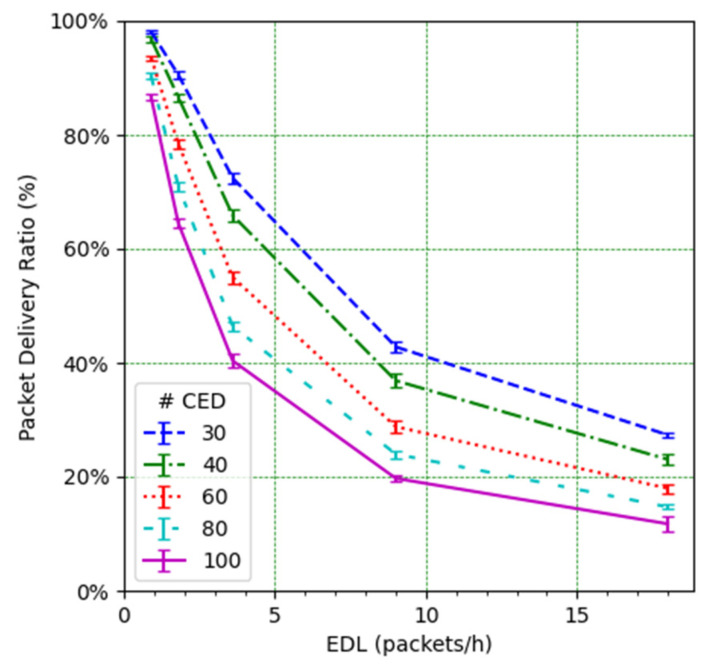
PDR for CEDs vs. EDL.

**Figure 12:**
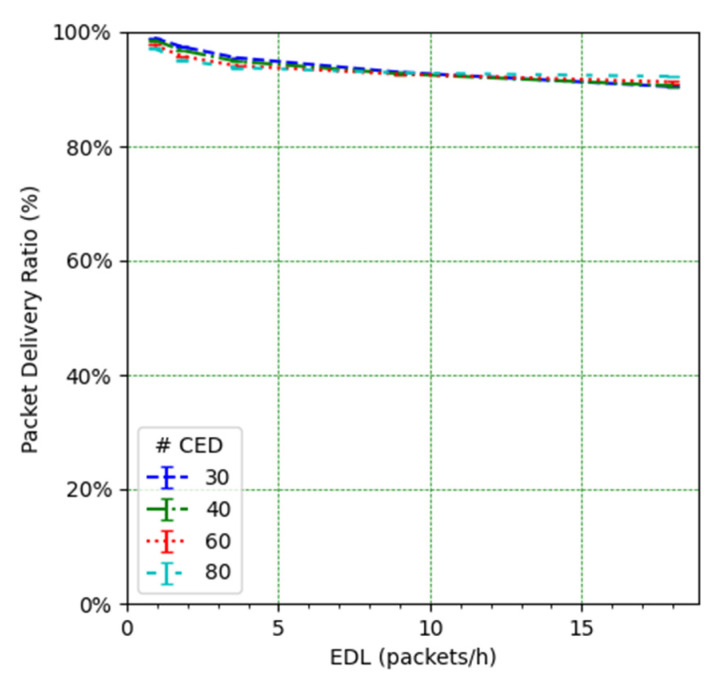
PDR for EDs in unconfirmed transmission mode vs. EDL.

We next study the impact of the EDL and the number of CEDs on the PDR (see Figure 10). It can be observed how such an impact is significant. For low EDL values, as the EDL increases, the PDR decreases quickly, especially for a high number of CEDs. However, the decrease slows down as the EDL increases. This behavior can be understood by looking at the PDR results corresponding to the CED and UED transmissions (see Figure 11 and Figure 12, respectively). While in the former the PDR exhibits a steady degradation with the EDL, the latter shows only a slight PDR decrease with the EDL, even for high EDL values.

Figure 11 also shows that the EDL has a dramatic effect on the PDR, while the number of CEDs generally has a lower influence on the PDR. However, for medium loads, the number of CEDs becomes more relevant. This result can be attributed to the higher impact of the unconfirmed transmissions at high loads when the number of CEDs is low.

On the other hand, the high PDR for unconfirmed transmissions shown in Figure 12 indicates that, in contrast with the CEDs, the UEDs are not affected by network congestion, even under a high UED load. Furthermore, the UEDs’ PDR is almost not influenced by the CED transmissions or by the number of CEDs. This behavior is due to the constant use of SF7 (i.e., the initial SF value for all the EDs in our considered scenarios) by the UEDs, which yields the lowest possible frame ToA.

**Figure 13:**
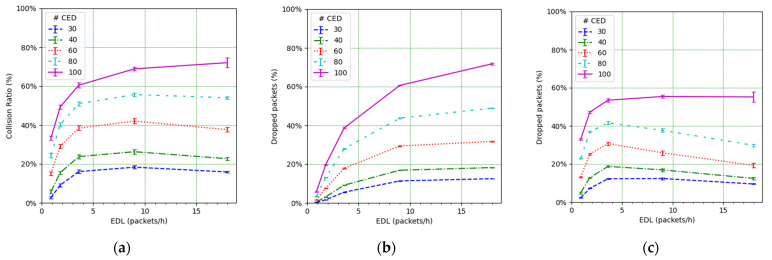
(**a**) Collision ratio at the gateway. (**b**) Packet drop ratio at EDs due to duty cycle restrictions. (**c**) Packet drop ratio at the gateway due to duty cycle restrictions.

In order to further analyze the reasons for the network PDR behavior, we next focus on the additional performance parameters. Figure 13 illustrates the number of MAC frame collisions over the total number of transmitted frames, along with the number of dropped packets due to duty cycle restrictions over the total number of packets intended to be transmitted by the EDs and by the gateway, respectively, in the considered scenarios. Note that in those scenarios, the downlink traffic forwarded by the gateway comprises only the ACKs sent by the NS. As load increases, the number of collided frames and dropped packets also increases quickly for low loads. However, for a high EDL, this increase slows down, even becoming a decrease, except for scenarios with 100 CEDs (see Figure 13a,c). There are two main reasons for this decrease. First, as the CEDs increase their SF, the frame ToA increases, therefore the number of duty cycle losses increases. Consequently, for high EDL the CED collisions do not increase as much as for the low EDL because there is a reduced number of CED transmissions. Second, the UED transmissions contribute a low number of collisions due to their use of SF7, as discussed earlier. On the other hand, the packet drops at the EDs affect the PDR for both types of EDs, while packet drops at the gateway have a negative effect, mainly on the CEDs’ PDR. All these behaviors have a great impact on the PDR (see Figure 10), leading to a PDR decrease with the EDL that slows down as the EDL increases. 

The observations from Figure 13 are also relevant to understand the possible impact of the number of channels on the SF12 Well problem. For a greater number of channels (e.g., 8 channels), a lower number of data frame collisions and ED packet drops, and thus a PDR increase, is expected. While the number of ED retries might appear to decrease as a result, thus delaying the SF12 Well problem, another consequence of the PDR increase is a greater number of ACKs to be sent by the gateway, increasing packet (i.e., ACK) drops at the gateway. The EDs awaiting the ACKs that will not be received will anyway perform retries, and increase their SF, for the corresponding packets (even if such packets have actually been successfully delivered).

## 5. End-Device-Based Solutions

The analysis in the previous section has shown that all the CEDs evolve to use SF12 at a given WFT in the scenarios considered. Thereafter, the EDs use the worst SF configuration in terms of network congestion. In this section, we propose three alternative SF management techniques based on simple changes to how the SF parameter is managed by the ED. Then, we present and discuss an evaluation of these solutions.

### 5.1. Proposed Solutions

As explained in Section 2.3, LoRaWAN devices typically increase the SF after two consecutive ACKs not received. This behavior will henceforth be referred to as SF Mode 0 (SFM0). As alternatives, we introduce three new SF parameter management techniques, namely SF Mode 1 (SFM1), SF Mode 2 (SFM2), and SF Mode 3 (SFM3). They are defined as follows:

1. SFM1: SF7, which corresponds to the greatest DR value, is always used. No SF change is conducted even if the data or the ACK frames are lost.
2. SFM2: this technique adds an extra step to SFM0. When the SF reaches the SF12 value, if the corresponding ACK is not received after two transmission opportunities, the SF is reset to SF7. The rationale for this approach is that after unsuccessful transmission using SF12 it may be better to switch to SF7 as congestion might be the reason for the frame losses. Hence, SFM2 leads to a cyclic use of all the SF values.
3. SFM3: the basis for this technique is also SFM0. However, with this technique if an ACK is received, the ED will decrease its SF value. Hence, this option brings the opportunity to increase the DR, with an expectation to reduce the frame ToA and thus reduce network congestion.

Note that we consider SFM1 as a benchmark for our simulation scenarios as it minimizes the frame ToA. Obviously, it cannot be considered a general solution (i.e., for any type of LoRaWAN network) because its performance will be severely affected for high numbers of CEDs, due to collisions, or if the radio link quality is not good enough.

### 5.2. Evaluation

In this subsection, we evaluate the performance of the presented alternative SF management techniques (i.e., SFM1, SFM2, and SFM3) in the same conditions considered for SFM0 in Section 4.

**Figure 14:**
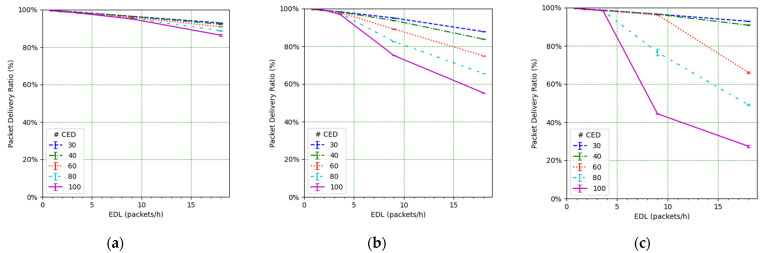
PDR vs. EDL for (**a**) SFM1, (**b**) SFM2, and (**c**) SFM3.

**Figure 15:**
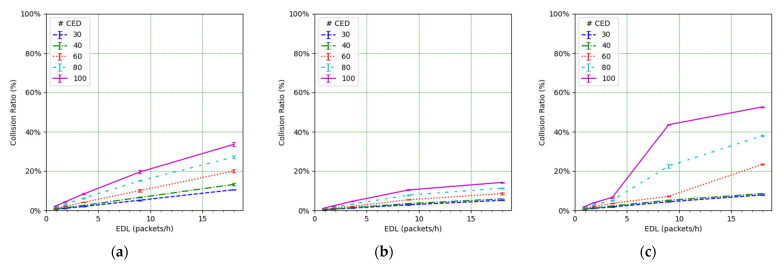
Frame collisions at the gateway vs. EDL for (**a**) SFM1, (**b**) SFM2, and (**c**) SFM3.

**Figure 16:**
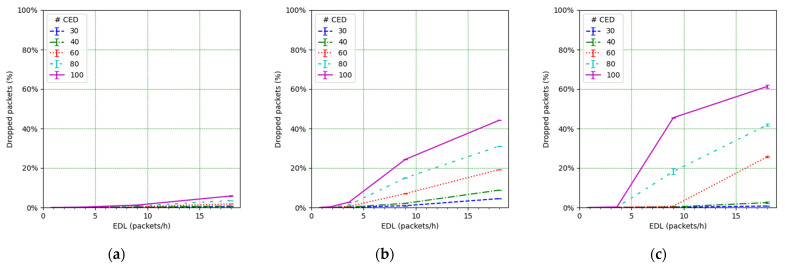
Packet losses due to duty cycle restrictions at the EDs vs. EDL for (**a**) SFM1, (**b**) SFM2, and (**c**) SFM3.

**Figure 17:**
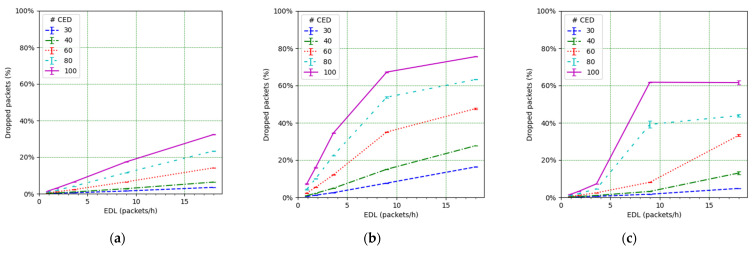
Packet losses due to duty cycle at the gateway vs. EDL for (**a**) SFM1, (**b**) SFM2, and (**c**) SFM3.

**Figure 18:**
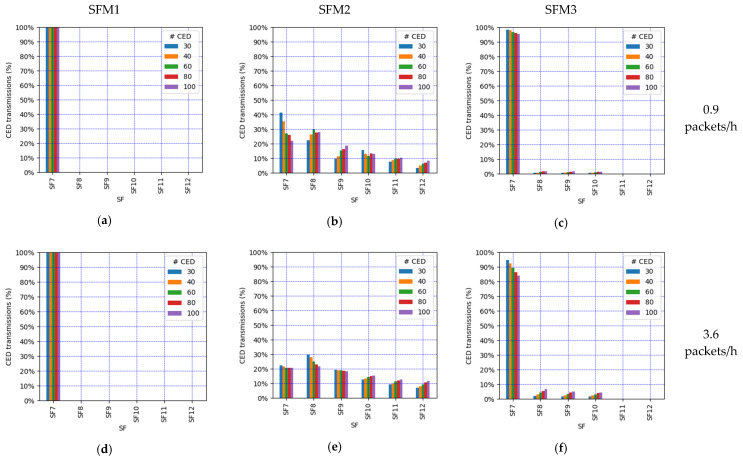

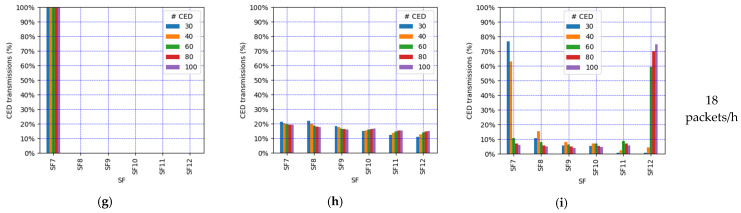
CED SF values distribution when SF mode is (**a**,**d**,**g**) SFM1; (**b**,**e**,**h**) SFM2; and (**c**,**f**,**i**) SFM3 and for (**a**–**c**) EDL equal to 0.9 packets/h, (**d**–**f**) 3.6 packets/h, and (**g**–**i**) 18 packets/h.

We focus on the network PDR as the main performance metric (Figure 14). However, in order to better understand the network performance and behavior, we also study the number of collisions (Figure 15), the number of losses due to duty cycle restrictions at both the ED (Figure 16) and the gateway (Figure 17), and the distribution of the SF values used for each mode, and for each considered number of CEDs (Figure 18). 

Figure 14 illustrates the total PDR for traffic including confirmed and not-confirmed transmission modes, as a function of the EDL. SFM1 (see Figure 14a) yields the highest PDR among the considered SF management modes, even in high load conditions. For SFM1, the PDR is always above 80% in the considered scenarios. SFM1 also offers the best behavior in terms of losses due to duty cycle restrictions (see Figure 16a and Figure 17a). This best performance under congestion is due to the fact that SF7 leads to the minimum ToA, which minimizes channel utilization and the impact of the duty-cycle limitation on network performance. This occurs despite the fact that the collision ratio of SFM1 is not the best among the considered SF management techniques, except for a very low packet load (see Figure 15). The observed collision ratio is a consequence of the approach in SFM1 based on always using the same SF value (i.e., SF7), which precludes exploiting the orthogonality that stems from using different SF values.

It is interesting to compare the high PDR achieved by using SFM1 (Figure 14a), with the low PDR obtained by the CEDs when using SFM0 (Figure 11) for the same range of EDL values and number of CEDs. The low PDR of SFM0 is mainly due to the use of high SF values (equal to SF12 for all CEDs after the WFT), which lead to high ToA values and produce a high number of collisions at the gateway (Figure 13a) and packet drops at the EDs (Figure 13b). The gateway is also unable to transmit all the ACKs to the corresponding CEDs due to duty cycle restrictions (Figure 13c), leading to unnecessary retries by those CEDs which increase the offered load and contribute to the decrease of the PDR. In contrast, in SFM1 all nodes use SF7, which minimizes the ToA and yields a high PDR.

However, we hypothesize that there exists a higher number of CEDs and/or traffic load that will create a low PDR problem equivalent to the one found for SFM0, regardless of the SF values used by the EDs.

In contrast with the SFM1 behavior, SFM3 leads to a quick PDR decrease as the offered load increases, especially for a high number of CEDs (Figure 14c). This is due to an increase in the number of CEDs that use SF12 or an SF value close to this one (see Figure 18i), leading to greater ToA values and contributing to higher collision probability (see Figure 15c), which results in low PDR values. On the other hand, for a low number of CEDs, the PDR obtained with SFM3 tends to be the highest among all of the considered SF management modes. This is, again, related to the distribution of the SF values used by the CEDs: they mainly use SF7 for a low number of CEDs, even for high EDL (see Figure 18i). This SF value distribution leads to a low collision ratio (see Figure 15c and a very low number of losses due to duty cycle restrictions, at both the EDs (see Figure 16c) and the gateway (see Figure 17c). Finally, SFM3 offers a higher PDR compared with SFM0 by a factor of up to 2.44, within the study conditions.

SFM2 leads to intermediate PDR results (see Figure 14b) because it produces a distribution of SF values used by the CEDs that is rather uniform (see Figure 18b,e,h). Only a significant SF value distribution difference exists for a very low EDL, but even in this case, the probability of using the most likely SF value (i.e., SF7) is below 41% for all numbers of the CEDs considered (see Figure 18b). The more even distribution of SF values achieved by SFM2 (see Figure 18h) exploits the orthogonality of different SF values and leads to less collisions than the rest of the considered SFMs, especially for a high load (see Figure 15). However, the number of losses due to duty cycle restrictions of SFM2 is greater than the one obtained for SFM1 (at both the CEDs and the gateway), and, for high EDL and a high number of CEDs, it is also greater than the SFM3 one, regarding losses at the gateway (see Figure 16b and Figure 17b, respectively). Such behavior of SFM2 is due to its tendency to lead to greater frame ToA, because of its greater probability of using greater SF values than those of SFM1 and SFM3 (regarding the latter, one exception is the high EDL and the high number of CEDs). Nevertheless, SFM2 yields a PDR increase, compared with SFM0 (Figure 10), by a factor of up to 4.7 for the range of scenarios considered.

On the other hand, comparing losses due to duty cycle restrictions at the gateway for SFM0, SFM2, and SFM3 schemes (Figure 13c and Figure 17b,c), we can appreciate an increase in those losses for the mentioned alternative SFMs. For SFM0, the lower number of packet (ACK) drops at the gateway is due to a lower PDR (which is due to a greater number of packet losses due to collisions, and the duty cycle restrictions at the EDs) and thus a lower number of ACKs to be sent. For the alternative SF management schemes, the PDR is greater than for SFM0, which increases the ACK traffic, and the number of packet drops at the gateway. Therefore, the gateway becomes a limitation for the alternative SF management techniques.

From the above analysis, it can be highlighted that any of the considered alternative SF management techniques allow the avoidance of the SF12 Well and improve the network PDR. SFM1 offers the best performance in terms of the PDR and packet losses due to duty cycle restrictions, although, as aforementioned, this SF management technique is only included in the evaluation as a benchmark. Both SFM2 and SFM3 outperform SFM0 but offer different trade-offs. In terms of collisions at the gateway, SFM2 is the best option because it presents a more even SF value distribution. However, for low loads, SFM3 offers a lower number of duty-cycle-induced packet drops, due to a higher fraction of devices using low SF values. In consequence, SFM2 tends to offer a greater PDR than SFM3 for high EDL and a high number of CEDs, whereas SFM3 yields a higher PDR than SFM2 for a low EDL and a low number of CEDs.

## 6. Conclusions

In this paper, we identified and characterized by simulation a formerly unexplored LoRaWAN network condition, which we call the SF12 Well. This phenomenon may arise due to the presence of even a relatively low number of CEDs, which will tend to increase their SF and thus the number of collisions and packet drops due to duty cycle constraints. In consequence, the SF12 Well may significantly degrade network performance, leading to a very low PDR. The negative impact of the SF12 Well on the PDR increases with the number of CEDs and the EDL.

We evaluated alternative ED-oriented SF management techniques which show that it is possible to avoid the SF12 Well, while achieving a significant PDR improvement compared with default LoRaWAN behavior by up to a factor of 4.7 in the scenarios considered. A crucial feature of alternative SF management techniques is their SF value distributions. Even SF distributions, as in SFM2, allow the exploitation of the orthogonality of different SF values and reduce the number of collisions. Approaches leading to uneven SF distributions, especially when low SF values are highly utilized, as tends to occur in SFM3, reduce the ToA to a greater extent. While decreasing ToA reduces the number of collisions and losses due to duty cycle limitations, it misses the opportunity to exploit the orthogonality of using different SF values.

The best alternative SF management technique depends on the specific characteristics of each scenario, particularly in terms of the number of CEDs and the EDL. The above remarks will offer helpful guidance for engineers or researchers who may need to face the SF12 Well problem.

## Figures and Tables

**Table 1:** DRs, SFs, and physical layer bit rates for the EU 868 MHz band channels.

Data Rate (DR)	Modulation	Spreading Factor	Bandwidth (kHz)	Bit Rate (bit/s)
0	LoRa	SF12	125	250
1	LoRa	SF11	125	440
2	LoRa	SF10	125	980
3	LoRa	SF9	125	1760
4	LoRa	SF8	125	3125
5	LoRa	SF7	125	5470
6	LoRa	SF7	250	11,000
7	GFSK			50,000

## Data Availability

Not applicable.

## References

[1] Raza U., Kulkarni P., Sooriyabandara M. (2017). Low Power Wide Area Networks: An Overview. IEEE Commun. Surv. Tutor..

[2] Chaudhari B.S., Zennaro M., Borkar S. (2020). LPWAN technologies: Emerging application characteristics, requirements, and design considerations. Future Internet.

[3] Minaburo A., Toutain L., Gomez C., Barthel D., Zuniga J.C. (2020). SCHC: Generic Framework for Static Context Header Compression and Fragmentation.

[4] Gomez C., Minaburo A., Toutain L., Barthel D., Zuniga J.C. (2020). IPv6 over LPWANs: Connecting low power wide area networks to the Internet (of Things). IEEE Wirel. Commun..

[5] Haxhibeqiri J., De Poorter E., Moerman I., Hoebeke J. (2018). A survey of LoRaWAN for IoT: From technology to application. Sensors.

[6] Augustin A., Yi J., Clausen T. (2016). A study of LoRa: Long range & low power networks for the Internet of Things. Sensors.

[7] Pop A.-I., Raza U., Kulkarni P., Sooriyabandara M. Does Bidirectional Traffic Do More Harm Than Good in LoRaWAN Based LPWA Networks?. Proceedings of the GLOBECOM 2017—2017 IEEE Global Communications Conference.

[8] Capuzzo M., Magrin D., Zanella A. Confirmed Traffic in LoRaWAN: Pitfalls and Countermeasures. Proceedings of the 2018 17th Annual Mediterranean Ad Hoc Networking Workshop (Med-Hoc-Net).

[9] Magrin D., Capuzzo M., Zanella A. (2020). A Thorough Study of LoRaWAN Performance Under Different Parameter Settings. IEEE Internet Things J..

[10] Lavric A., Popa V. (2018). Performance Evaluation of LoRaWAN Communication Scalability in Large-Scale Wireless Sensor Networks. Wirel. Commun. Mob. Comput..

[11] Böcker S., Arendt C., Jörke P., Wietfeld C. LPWAN in the Context of 5G: Capability of LoRaWAN to Contribute to MMTC. Proceedings of the 2019 IEEE 5th World Forum on Internet of Things (WF-IoT).

[12] FLoRa Framework for LoRa. https://flora.aalto.fi.

[13] AFLoRa Advanced Framework for LoRaWAN. https://github.com/lluiscas/AFloRa.

[14] Casals L., Mir B., Vidal R., Gomez C. (2017). Modeling the Energy Performance of LoRaWAN. Sensors.

[15] LoRa Alliance Technical Committee (2020). LoRaWAN® L2 1.0.4 Specification (TS001-1.0.4).

[16] Semtech Corporation (2013). SX1272/3/6/7/8: LoRa Modem. Designer’s Guide. AN1200.13.

[17] LoRa Alliance Technical Committee (2020). LoRaWAN™ 1.0.2 Regional Parameters.

[18] ETSI EN 300.220-2 v3.2.1 (2018-06) Short Range Devices (SRD) Operating in the Frequency Range 25 MHz to 1000 MHz; Part 2: Harmonised Standard for Access to Radio Spectrum for Non Specific Radio Equipment. https://www.etsi.org/deliver/etsi_en/300200_300299/30022002/03.02.01_60/en_30022002v030201p.pdf.

[19] LoRa Alliance Technical Committee (2018). LoRaWAN Specification.

[20] LoRa Alliance Certification. https://lora-alliance.org/lorawan-certification.

[21] Capuzzo M., Magrin D., Zanella A. Mathematical Modeling of LoRa WAN Performance with Bi-Directional Traffic. Proceedings of the 2018 IEEE Global Communications Conference (GLOBECOM).

[22] Mikhaylov K., Petäjäjärvi J., Pouttu A. Effect of Downlink Traffic on Performance of LoRaWAN LPWA Networks: Empirical Study. Proceedings of the 2018 IEEE 29th Annual International Symposium on Personal, Indoor and Mobile Radio Communications (PIMRC).

[23] Di Vincenzo V., Heusse M., Tourancheau B. Improving Downlink Scalability in LoRaWAN. Proceedings of the ICC 2019-2019 IEEE International Conference on Communications (ICC).

[24] Sanchez-Iborra R., Sanchez-Gomez J., Ballesta-Viñas J., Cano M.-D., Skarmeta A.F. (2018). Performance Evaluation of LoRa Considering Scenario Conditions. Sensors.

[25] Hasegawa Y., Suzuki K. A Multi-User ACK-Aggregation Method for Large-Scale Reliable LoRaWAN Service. Proceedings of the ICC 2019-2019 IEEE International Conference on Communications (ICC).

[26] Kim B., Hwang K.-i. (2017). Cooperative Downlink Listening for Low-Power Long-Range Wide-Area Network. Sustainability.

[27] LoRa Alliance Technical Committee (2017). LoRaWAN Specification.

[28] Kufakunesu R., Hancke G.P., Abu-Mahfouz A.M. (2020). A Survey on Adaptive Data Rate Optimization in LoRaWAN: Recent Solutions and Major Challenges. Sensors.

[29] Garlisi D., Tinnirello I., Bianchi G., Cuomo F. (2021). Capture Aware Sequential Waterfilling for LoRaWAN Adaptive Data Rate. IEEE Trans. Wirel. Commun..

[30] Reynders B., Meert W., Pollin S. Power and Spreading Factor Control in Low Power Wide Area Networks. Proceedings of the 2017 IEEE International Conference on Communications (ICC).

[31] Sallum E., Pereira N., Alves M., Santos M. (2020). Improving Quality-Of-Service in LoRa Low-Power Wide-Area Networks through Optimized Radio Resource Management. J. Sens. Actuator Netw..

[32] Bankov D., Khorov E., Lyakhov A. (2019). LoRaWAN Modeling and MCS Allocation to Satisfy Heterogeneous QoS Requirements. Sensors.

[33] Slabicki M., Premsankar G., Di Francesco M. Adaptive Configuration of Lora Networks for Dense IoT Deployments. Proceedings of the NOMS 2018-2018 IEEE/IFIP Network Operations and Management Symposium.

[34] Kim D.-Y., Kim S., Hassan H., Park J.H. (2017). Adaptive Data Rate Control in Low Power Wide Area Networks for Long Range IoT Services. J. Comput. Sci..

[35] Cattani M., Boano C.A., Römer K. (2017). An Experimental Evaluation of the Reliability of LoRa Long-Range Low-Power Wireless Communication. J. Sens. Actuator Netw..

[36] Mikhaylov K., Stusek M., Masek P., Fujdiak R., Mozny R., Andreev S., Hosek J. On the Performance of Multi-Gateway LoRaWAN Deployments: An Experimental Study. Proceedings of the 2020 IEEE Wireless Communications and Networking Conference (WCNC).

[37] Li L., Ren J., Zhu Q. On the Application of LoRa LPWAN Technology in Sailing Monitoring System. Proceedings of the 2017 13th Annual Conference on Wireless On-demand Network Systems and Services (WONS).

[38] Di Renzone G., Parrino S., Peruzzi G., Pozzebon A., Bertoni D. (2021). LoRaWAN Underground to Aboveground Data Transmission Performances for Different Soil Compositions. IEEE Trans. Instrum. Meas..

[39] Alves H.B.M., Lima V.S.S., Silva D.R.C., Nogueira M.B., Rodrigues M.C., Cunha R.N., Carvalho D.F., Sisinni E., Ferrari P. Introducing a Survey Methodology for Assessing LoRaWAN Coverage in Smart Campus Scenarios. Proceedings of the 2020 IEEE International Workshop on Metrology for Industry 4.0 IoT.

